# Seronegative enthesoarthritis as the first presentation of the atrial myxoma

**DOI:** 10.2478/rir-2023-0024

**Published:** 2023-09-27

**Authors:** Bonomi Francesco, Orlandi Martina, Conforti Maria Letizia, Guiducci Serena, Matucci Cerinic Marco

**Affiliations:** Department of Clinical and Experimental Medicine, University of Florence, 50134 Florence, Italy; Unit of Immunology, Rheumatology, Allergy and Rare Diseases (UnIRAR), IRCCS San Raffaele Hospital, 20132 Milan, Italy

**Keywords:** arthritis, atrial myxoma, paraneoplastic syndromes, vasculitis

## Abstract

Atrial myxoma (AM) is the most common primary cardiac tumor. Its clinical presentation can be highly heterogeneous and can be characterized by many constitutional manifestations and development of rheumatologic symptoms.We report the case of a patient presenting with a seronegative arthritis characterized by articular and enthesis involvement and purpuric cutaneous lesions that was refractory to conventional treatments and that was later diag- nosed with an AM as first cause of the manifestations. AM can present with different symptoms; among them, it is able to cause some rheumatological manifestation as it is able to secrete proinflammatory cytokines, as interleukin 6 (IL-6), tumor necrosis factor α (TNF-α), and interferon γ (IFN-γ). The present case is of particular interest as it presents an AM as the cause of an inflammatory arthropathy with articular and enthesis involvement. A paraneoplastic screening is always relevant in rheumatology, especially when encountering a refractory disease.

## Introduction

Atrial myxoma (AM) is a rare, but the most common primary cardiac tumor. The clinical presentation depends on the dimension and localization of the mass and includes embolism, intracardiac obstruction (episodes of dyspnea, orthopnea), and constitutional systemic manifestations (fever, arthralgias, weight loss).^[1]^ The release of inflammatory cytokines by the tumor can lead to paraneoplastic syndromes and, in some cases, to rheumatologic manifestations. Blood tests may show anemia and increase in inflammatory indices such as erythrocyte sedimentation rate (ESR) or C-reactive protein (CRP).^[2]^ Diagnosis is usually suggested by transthoracic (TTE) or transesophageal (TEE) echocardiography that also allows determination of the location, size, shape, invasiveness, and mobility of the tumor preoperatively.^[3]^ Biopsy is usually not performed since AM has a high risk of embolization. Treatment is surgical with a high success rate.^[4]^

We present a case of an AM that presented as a seronegative enthesoarthritis with purpuric cutaneous lesions as it is fundamental, for rheumatologists, to keep in mind the possible differential diagnosis and necessity of performing paraneoplastic screening when facing a rheumatic disease of new onset, especially when it is scarcely responsive to standard treatments.

**Figure 1:**
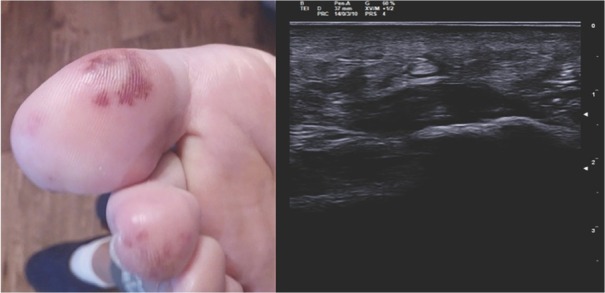
Purpuric lesions on the plantar side of the right foot (left). Echographic image of plantar fascia edema of the left foot (transverse section) (right).

A 31-year-old woman was admitted becayse of relapse of seronegative enthesoarthritis. The patient complained of arthralgias at metatarsophalangeal joints (MTP) bilaterally and pain on the plantar surface of the feet for 1 year, which showed a partial response to anti-inflammatory drugs. After 2 months from the onset of the articular symptoms, the patient developed purpuric lesions on the plantar surface bilaterally (Figure 1, left). No improvement happened after being treated with prednisone 50mg/d (rapidly tapered). Blood tests showed increased levels of inflammatory indices (CRP 12 mg/dL, and ESR 22 mm/h), negative for rheumatoid factor (RF), and anticitrullinated peptide autoantibodies (aCCP). Musculoskeletal ultrasound showed a bilateral plantar fascia edema (compatible with plantar fasciitis) and synovitis of right metacarpophalangeal joints (MTP) (Figure 1, right). Diagnosis of seronegative “early arthritis” was made, and the patient started treatment with methylprednisolone 4 mg/d and methotrexate (MTX) 7.5 mg/week, without improvement. The purpuric lesions and arthralgias worsened with onset of moderate exertional dyspnea and episodes of palpitations. No swollen joints was found during physical examination but diffuse MTP tenderness and pain at pressure at the plantar fascia bilaterally. Purpuric lesions were found on the plantar surface bilaterally. No heart murmur was detected. She underwent a blood test with evidence of increased inflammatory indices (ESR 52 mm/h, CRP 17 mg/dL), negative for antinuclear autoantibodies (ANA), extractable nuclear antigens (ENA), anti-dsDNA, anti-neutrophil cytoplasmic antibodies, antiphospholipid antibodies, cryoglobulins, and serology for HBV, HCV, tuberculosis, and syphilis. A nail videocapillaroscopy was in normal pattern. To better characterize the dyspnea, a TTE was performed which revealed a pedunculated mass in the left atrium.

**Figure 2:**
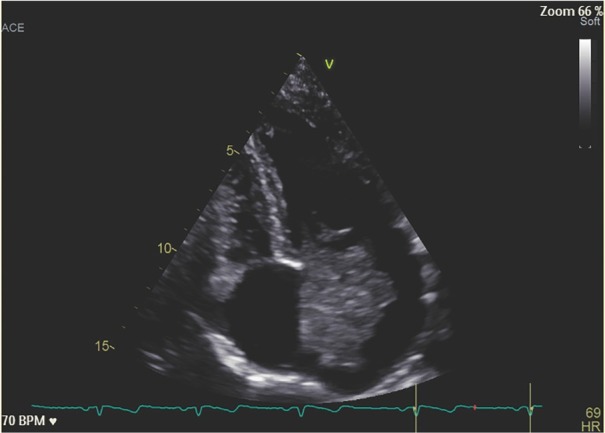
Echocardiographic image of the AM occupying almost all the left atrium and mitral valve. AM, atrial myxoma.

The mass was 4223 mm in diameter compatible with an AM (Figure 2). The patient was operated with the mass removed. After surgery, the patient reported a complete resolution of the articular and cutaneous symptoms and methylprednisolone and MTX were withdrawn.

## Discussion

As reported in the literature, cutaneous and articular symptoms may be possible paraneoplastic manifestations.^[5]^ Paraneoplastic arthritis (PA) usually do not respond to corticosteroids or conventional antirheumatic drugs, but encounter complete resolution after oncologic treatment.^[6]^ Usually, PA presents as abrupt onset of symptoms with elevated acute phase reactants, and negative for RF, aCCP, and ANA. AM is a rare cardiac tumor that can produce inflammatory cytokines (*e.g.*, interleukin 1 [IL-1], interleukin 6 [IL-6], tumor necrosis factor α [TNF-α], interferon γ [IFN-γ])^[7]^ fostering the growth of the tumor and the development of inflammatory symptoms (*e.g.*, low grade fever, weight loss, arthralgia). Constitutional symptoms are mainly attributed to the increased release of IL-6.^[8]^ Usually, IL-6 is involved in the increase of CRP, in tumor growth and recurrence and development of autoimmune features.^[7]^ The release of inflammatory cytokines is also related to the development of manifestations as Raynaud’s phenomenon, malar rash, and cutaneous vasculitis.^[9]^ However, this is the first case report to describe an enthesoarthritis as a manifestation of an AM. These features usually disappeared after AM removal but in some patients persistent elevation of the ANA titer was observed.^[9]^ Following excision, the IL-6 levels return to normal, and the systemic symptoms eventually disappear with normalization of IL-6.^[10]^ Cutaneous manifestations are seen in one-third of cases and are usually either due to embolization of the tumor mass or development of autoimmune features due to interleukins’ secretion. Embolization often leads to purpuric lesions on the palmar/plantar surfaces, telangiectasia, petechiae, or livedo reticularis.^[9]^

## Conclusions

The present case is of particular interest as it presents an AM as the cause of an inflammatory arthropathy with articular and enthesis involvement. Moreover, it highlights the importance of performing a thorough paraneoplastic screening when encountering a new onset arthritis, especially when it occurs in young patients and respond poorly to conventional treatments in patients of young age.
